# Diverse Trajectories of Hikikomori Symptoms During Job Search and the Role of Identity Distress: Three Wave Longitudinal Research

**DOI:** 10.3389/fpsyt.2022.897806

**Published:** 2022-07-07

**Authors:** Shogo Hihara, Kohei Kambara, Tomotaka Umemura, Kyonosuke Handa, Kazumi Sugimura

**Affiliations:** ^1^Faculty of Business Administration, Matsuyama University, Ehime, Japan; ^2^Faculty of Psychology, Doshisha University, Kyoto, Japan; ^3^Department of Psychology, Graduate School of Humanity and Social Sciences, Hiroshima University, Hiroshima, Japan

**Keywords:** hikikomori, trajectories, identity distress, Japan, longitudinal, young people, job search

## Abstract

**Objective:**

Hikikomori, a prolonged form of social withdrawal, has received attention in various research areas. This longitudinal study aimed to identify diverse trajectories of hikikomori symptoms among young Japanese adults engaged in a job search. It also tested whether identity distress, a critical developmental issue, predicts these trajectories while controlling for other risk factors (depressive symptoms, life satisfaction, career expectations, and gender).

**Methods:**

A total of 756 third-year Japanese university students (at Time 1, *M*_age_ = 20.88 years; women: 78.97%) who engaged in job search participated in our three-wave longitudinal survey at six-month intervals. To assess hikikomori symptoms, we used the 25-item Hikikomori Questionnaire. In addition, identity distress was measured using the 10-item Identity Distress Survey.

**Results:**

Latent class growth analysis revealed four different trajectories of hikikomori symptoms. Most young adults showed severe levels and escalating hikikomori symptoms over time. In contrast, a small proportion of young adults prevented hikikomori symptoms through the period of job search. Additionally, young adults with more severe levels of identity distress followed trajectories marked by severe hikikomori symptoms after controlling for other risk factors.

**Conclusion:**

The present study's findings contribute to developing a primary intervention for hikikomori symptoms by identifying the period of greatest risk. Group-based counseling support for hikikomori from the perspective of identity is recommended.

## Introduction

In recent decades, pathological social withdrawal, or “hikikomori,” has received attention in diverse fields, including psychiatry, psychology, and public health (1, 2). Hikikomori refers to a state of withdrawing from society and staying at home for 6 months or longer (3). Recent researchers have also proposed more strict diagnostic criteria for hikikomori, which requires: (a) physical withdrawal; (b) lack of social participation and interactions for 6 months or longer; and (c) distress related to social isolation (4). Furthermore, a novel self-rated questionnaire (the 25-item Hikikomori Questionnaire; HQ-25) was also developed to assess these symptoms (5). Withdrawing from society is seen in other psychiatric disorders, such as major depressive disorder (6). However, a prior study (7) indicated that almost half (45.5%) of hikikomori people do not show comorbid psychiatric disorders. Additionally, there are case studies that report those who withdraw because of trying to avoid failure in the future, not because of depression (8). Thus, hikikomori symptoms are a significant phenomenon that is not fully explained using other psychiatric disorders.

Epidemiologic surveys have reported that 1–2% of Japanese young people show a hikikomori condition (7). Furthermore, three extensive surveys conducted by the Cabinet Office of the Government of Japan in 2010, 2016, and 2019 have suggested the escalation of prevalence of hikikomori (9–11). Prevalence of hikikomori has also been found in a number of other countries, such as South Korea, Hong-Kong, and Italy (12–14). Hikikomori damages the economy, education in society, and individual mental health (1). Therefore, evaluating how hikikomori risks change over time and identifying crucial factors that predict these changes are required for beneficial interventions (15).

Late adolescence and young adulthood are vulnerable periods when many hikikomori cases first occur symptomatically (7, 8). Many young Japanese people seek jobs to enter the labor market during these periods. However, due to changes in social contexts and cultural values during the past several decades, young Japanese people have difficulties finding employment. For social shifts, economic depression, casualization of employment, and the collapse of a well-established seniority wage system have occurred in Japan (16). Regarding cultural shifts, increased globalization has diminished the power of Japan's traditional collectivism and has emphasized Western individualism (17). Such a change has rendered young Japanese people unable to rely on models or support from previous generations. Due to these social and cultural shifts, young Japanese people tend to have problems in (re)entering to the labor market. Furthermore, the educational system in Japan is single track that is rigidly organized and highly pressured (18). Traditionally, schools in Japan have strong connection with employers, and teacher recommendations serve an important role as a determinant of occupation. Although the number of high-quality jobs has decreased in several decades, teachers still have crucial job placement functions. Hence, Japanese young people and their parents recognize the importance of educational success and the lack of second chances. Additionally, the environments of Japanese schools are strictly controlled, and non-conformist behaviors are rarely tolerated. Amid these unstable and high-pressure contexts, a certain number of young people find it challenging to navigate their engagement in society successfully, and as a result, people may withdraw from society to avoid such stressful situations.

In such a risky situation in Japan, some young people can guide their job search successfully while preventing or modifying their hikikomori symptoms, whereas others cannot deal with this task while demonstrating severe hikikomori symptoms. These diverse trajectories of hikikomori symptoms have not been empirically explored to date, although cross-sectional research has been conducted (7). Revealing adaptive and maladaptive trajectories of hikikomori symptoms provides significant insights into how many young people need support and when they need it, consequently contributing to further understanding of the risk of hikikomori.

Previous studies have identified various risk factors for hikikomori. Depression is one of the most critical psychiatric conditions related to hikikomori symptoms (6, 7, 15, 19). From a psychological perspective, a lack of subjective well-being leads to hikikomori symptoms (20). Furthermore, some young people withdraw from society to avoid stressful life events (1, 8). As finding a job is crucial in late adolescence and young adulthood, individuals lacking positive expectations of the process experience stress and avoid failure by showing hikikomori symptoms. From a sociodemographic viewpoint, men are more likely to show severe hikikomori symptoms than women (9).

Despite insights into such risk factors, developmental factors have been relatively neglected in empirical studies (21). The developmental crisis in each stage involves both vulnerability and the potential for positive change, and successful resolution of that leads to lifelong adjustment (22). Hence, interventional approaches based on developmental factors may prevent hikikomori and establish subsequent adaptive pathways (23, 24).

This study focuses on identity, which is a crucial developmental task in adolescence and young adulthood (22). Identity represents a sense of self that is coherent across time (past, present, and future) and is accepted in sociocultural contexts. Young people develop their identity through integrating past, present, and future selves in a coherent manner and by aligning their identity with sociocultural expectations (25, 26). Some young people can achieve these tasks, but others fail and display severe identity distress (27). Identity distress refers to subjective distress regarding an inability to reconcile aspects of the self into a relatively coherent and acceptable sense of self (28). Both the Diagnostic and Statistical Manual of Mental Disorders (DSM)-5 and the International Classification of Diseases (ICD)-10 regard such identity problems as having a crucial role in various types of personality disorders (29, 30). Furthermore, severe identity distress leads to other psychopathologies, such as anxiety and antisocial behaviors (31, 32). Because identity serves as a rudder for directing one's life trajectories (33), those with severe identity distress may postpone their career choices and withdraw from society to re-establish their life direction (18). Although several studies have indicated relations between identity and hikikomori symptoms (34, 35), no research has examined whether identity distress longitudinally predicts heterogeneous trajectories of hikikomori symptoms.

This study aimed to extract diverse trajectories of hikikomori symptoms and investigate how these trajectories are predicted by identity distress. First, we explored whether various patterns of hikikomori symptoms manifest among young Japanese adults during the job search phase. We hypothesized that most young adults would follow a trajectory marked by either consistently weak or decreasing hikikomori symptoms. However, we also expected some young adults to deviate from such trajectories by showing either consistently severe or increased hikikomori symptoms. Second, we examined the relationship between identity distress and trajectories of hikikomori symptoms. We hypothesized that those with severe identity distress would follow trajectories marked by severe levels of or exacerbated hikikomori symptoms after controlling for other risk factors reported in prior studies, such as psychiatric (depressive symptoms), psychological (life satisfaction and career expectation), and sociodemographic (gender) factors.

## Materials and Methods

### Participants and Procedure

Participants included a community sample of young adults recruited for a five-wave longitudinal research project conducted in November 2019, February 2020, May 2020, August 2020, and November 2020. In line with an assessment of hikikomori symptoms that refers to the symptoms in the past 6 months, the present study used data from November 2019 (Time 1: T1), May 2020 (Time 2: T2), and November 2020 (Time 3: T3). The participants were registrants of an online research company in Japan, MyVoice Communication (https://www.myvoice.co.jp/), which has survey panels from approximately 50 countries worldwide. At T1, all participants were third-year university students planning to obtain a job after graduation. In Japan, most high school students (82.8%) pursue higher education (e.g., university and junior college), and the proportion of those who enter university is the highest among them (53.7%) (36). Furthermore, due to the simultaneous recruitment of new graduates in Japan, many third-year university students search for jobs. Thus, our sample represents a large proportion of young adults engaged in job searches. Participants received an e-mail including (a) an explanation of the study aim and (b) a hyperlink to complete the online survey. Those who provided informed consent answered at T1 (*n* = 756), T2 (*n* = 266), and T3 (*n* = 170). This study was approved by the Ethics Review Board of Hiroshima University in Japan.

Table 1 reports the demographic characteristics of the present data. At T1, the age range of participants was 20–22 years (*M* = 20.88; *SD* = 0.64). Among them, 21.03% were men, and 78.97% were women. The sample was diverse regarding the parents' educational backgrounds and geographic regions. Regarding participants' parents' educational background, 61.64% of fathers and 64.81% of mothers had completed higher education. Regarding region, 75.79% and 24.21% of participants lived in relatively urban (Kanto, Chubu, and Kinki districts) and rural areas (Hokkaido, Tohoku, Chugoku, Shikoku, and Kyushu districts), respectively.

**Table 1:** Demographic information of the present sample.

**Demographic characteristics**	**Detail**	**Number (%)**		
		**T1 (*n* = 756)**	**T2 (*n* = 266)**	**T3 (*n* = 170)**
Gender	Men	159 (21.03)	48 (18.05)	23 (13.53)
	Women	597 (78.97)	218 (81.95)	147 (86.47)
Age	20	203 (26.85)	—	—
	21	440 (58.20)	—	—
	22	113 (14.95)	—	—
Father's educational level	Secondary school	232 (30.69)	86 (32.33)	60 (35.29)
	Higher education	466 (61.64)	164 (61.65)	96 (56.47)
	Missing	58 (7.67)	16 (6.02)	14 (8.24)
Mother's educational level	Secondary school	229 (30.29)	111 (41.73)	67 (39.41)
	Higher education	490 (64.81)	145 (54.51)	94 (54.29)
	Missing	37 (4.89)	10 (3.76)	9 (5.29)
Region	Hokkaido (North part)	63 (8.33)	3 (1.13)	3 (1.76)
	Tohoku (Northeast part)	115 (15.21)	18 (6.77)	7 (4.12)
	Chubu (Middle part)	106 (14.02)	34 (12.78)	25 (14.71)
	Kanto (Middle east part)	203 (26.85)	108 (40.61)	65 (38.24)
	Kinki (Middle west part)	177 (23.41)	60 (22.56)	50 (29.41)
	Chugoku (West part)	113 (14.95)	16 (6.02)	8 (4.71)
	Shikoku (Southwest part)	14 (1.85)	4 (1.50)	3 (1.76)
	Kyushu (South part)	121 (16.01)	23 (8.65)	9 (5.29)

*T, time*.

The type of missingness was examined in two steps. First, we conducted Little's test of missing completely at random (37). The test indicated an insignificant result (χ^2^ (2,767) = 2,419.38, *p* = 1.000), suggesting that the missing pattern may be completely at random. Second, we compared participants who did or did not answer at T2 and T3 regarding the scores of the study variables at T1 using a series of *t*-tests. Participants who answered at T2 [*t* (754) = 2.46, *p* = 0.014, *d* = 0.19] and T3 [*t* (754) = 2.50, *p* = 0.013, *d* = 0.22] scored higher on hikikomori symptoms than those who did not answer at T2 and T3, respectively. Chi-square tests revealed that women were more likely to participate in T3 than men [χ^2^ (1, *N* = 756) = 7.43, *p* = 0.006, Cramer's *V* = 0.10]. However, all these effect sizes were small. Thus, we used full information maximum likelihood estimation to address missingness.

### Measures

#### Hikikomori Symptoms

We used the HQ-25 (5). This scale includes 25 items assessing the presence of hikikomori's psychological and behavioral characteristics over the past 6 months, such as lack of social connection and withdrawn behavior (sample item is “I shut myself in my room”). Each item is coded from 0 (strongly disagree) to 4 (strongly agree). We calculated the total score, ranging from 0 to 100. A previous clinical study using this measurement proposed a cutoff score of 42, which identifies individuals diagnosed with hikikomori (5). Cronbach's alpha reliability ranged from 0.92–0.93 across T1–T3.

#### Identity Distress

Identity distress was evaluated using the Identity Distress Survey (27, 38). This scale comprises ten items. The initial seven items measure the degree to which participants experience identity distress in seven life domains (e.g., long-term goals, career choice, and friendships). The remaining three items assess global severity, impact, and length of identity distress in these seven domains. Each item was coded using a 5-point Likert scale ranging from 1 (not at all) to 5 (very severely). In line with prior studies [e.g., Berman et al. (27)], we calculated the total score of the initial seven items, ranging from 7 to 35. Cronbach's alpha reliability was 0.77.

#### Depressive Symptoms

We used the Center for Epidemiological Studies Depression Scale (39, 40). This scale includes 20 questions about the presence of depressive symptoms over the previous 2 weeks (sample item: “I was bothered by things that usually don't bother me”), each coded from 1 (never or rarely) to 3 (most or all of the time). The total score ranges from 20 to 80. Cronbach's alpha reliability was 0.88.

#### Life Satisfaction

We used the Satisfaction with Life Scale (41). It includes five items assessing the degree to which participants have a sense of satisfaction with their life as a whole (sample item: “In most ways, my life is close to my ideal”). Each item was rated on a 7-point scale, ranging from 1 (completely untrue) to 7 (completely true). The scores for life satisfaction were summed to calculate the total score, which ranged from 5 to 35 points. Cronbach's alpha reliability was 0.89.

#### Career Expectation

We utilized four items measuring participants' positive expectations that their career decision-making behaviors would be effective for subsequent career decisions (sample item: “If I spend enough time gathering information about careers, I can learn what I need to know to make a good decision”) (42, 43). Participants rated each item on a 5-point Likert scale, ranging from 1 (strongly disagree) to 5 (strongly agree). We calculated the total score for career expectations by summing the four-item scores, which ranged from 5 to 20. Cronbach's alpha reliability was 0.88.

### Statistical Analyses

The statistical analyses employed SPSS Version 25 and Mplus Version 8.3 (44, 45). To identify diverse trajectories of hikikomori symptoms in the sample, we estimated latent class growth analysis (LCGA) (46). While latent growth curve modeling estimates a single average intercept and slope as well as their variance in the entire sample (47), LCGA allows for multiple latent intercepts and slopes, which explains the level and change patterns for the sample subgroups. The LCGA classifies participants into subgroups based on homogeneity and heterogeneity in intercepts and slopes. Therefore, this procedure is appropriate for identifying the diverse trajectories of hikikomori symptoms. We estimated different models, ranging from one-to six-class models. We define the number of classes based on these three criteria. First, we employed the sample-size adjusted Bayesian information criterion (SSA-BIC) (48). A smaller value of SSA-BIC with *k* class indicates that the model fits the data better than that with the *k* – 1 class. Second, we conducted the Lo–Mendell–Rubin likelihood ratio test (LMR-LRT) (49), which compares model fit in the k class with that in the *k* – 1 class. The test's significant result (*p* < 0.05) indicates that the *k*-class model is a better fit. Third, we evaluated entropy, which represents a standardized measure for classifying individuals based on their posterior class probabilities. The value of entropy can range from 0 to 1, and values above 0.70 indicate good classification accuracy (50). Additionally, considering that the class solutions depend on the sample, we tested their stability using double-split cross-validation. Specifically, we randomly split the entire sample into two subsamples and repeated the LCGA for each. We then used kappa (κ) coefficients to examine whether the subsamples' class designations were consistent with the entire sample's class designations. For ancillary analysis, to further clinical insights, we calculated the mean percentage of time points where hikikomori scores exceeded the cutoff point across T1–T3 for each trajectory.

To test the associations of identity distress with trajectories of hikikomori symptoms, we used multinomial logistic regression analysis. The explanatory variables were identity distress, depressive symptoms, life satisfaction, career expectations, and gender, whereas the objective variables were latent classes of hikikomori trajectories. Gender was included as a dummy variable (0 = men; 1 = women). We reported odds ratios (ORs) and their 95% confidence intervals. Additionally, we used a multiple regression analysis, including identity distress and other predictors as explanatory variables. The objective variable was the percentage of time points when participants' hikikomori symptom scores exceeded the cutoff score across T1–T3. All tests were two-tailed; *p*-values < 0.05 were regarded statistically significant.

## Results

### Preliminary Analyses

Table 2 presents descriptive statistics of the study variables. Table 3 shows the bivariate correlations between the study variables. Identity distress was positively correlated with hikikomori symptoms across T1–T3 (*r*s = 0.16–0.34).

**Table 2:** Descriptive statistics of the study variables.

**Variables**	**Time**	**Observed range**	***Min***	***Max***	***M***	***SD***	***Skewness***	***Kurtosis***
Hikikomori symptoms	T1	2–99	0	100	41.40	17.52	0.16	0.08
	T2	2–100	0	100	47.04	17.07	−0.05	0.48
	T3	1–97	0	100	48.05	16.86	0.16	1.14
Identity distress	T1	7–35	7	35	18.09	5.17	0.13	0.15
Depressive symptoms	T1	20–77	20	80	39.87	10.09	0.62	0.01
Life satisfaction	T1	5–35	5	35	19.32	6.51	−0.14	−0.11
Career expectations	T1	4–20	4	20	14.78	3.25	−0.73	0.93

*T, time; Min, minimum value; Max, maximum value; M, mean; SD, standard deviation*.

**Table 3:** Bivariate correlations between predictors at T1 and hikikomori symptoms across T1–T3.

**Predictors at T1**	**Hikikomori symptoms**		
	**T1**	**T2**	**T3**
Identity distress	0.34^***^	0.25^***^	0.16^*^
Depressive symptoms	0.51^***^	0.49^***^	0.37^***^
Life satisfaction	−0.39^***^	−0.36^***^	−0.32^***^
Career expectation	−0.25^***^	−0.17^**^	−0.15^*^
Gender (0 = men; 1 = women)	−0.07^*^	−0.07	−0.00

*T, time*.

* *p < 0.05*,

** *p < 0.01*,

*** *p < 0.001*.

### Diverse Trajectories of Hikikomori Symptoms

**Figure 1:**
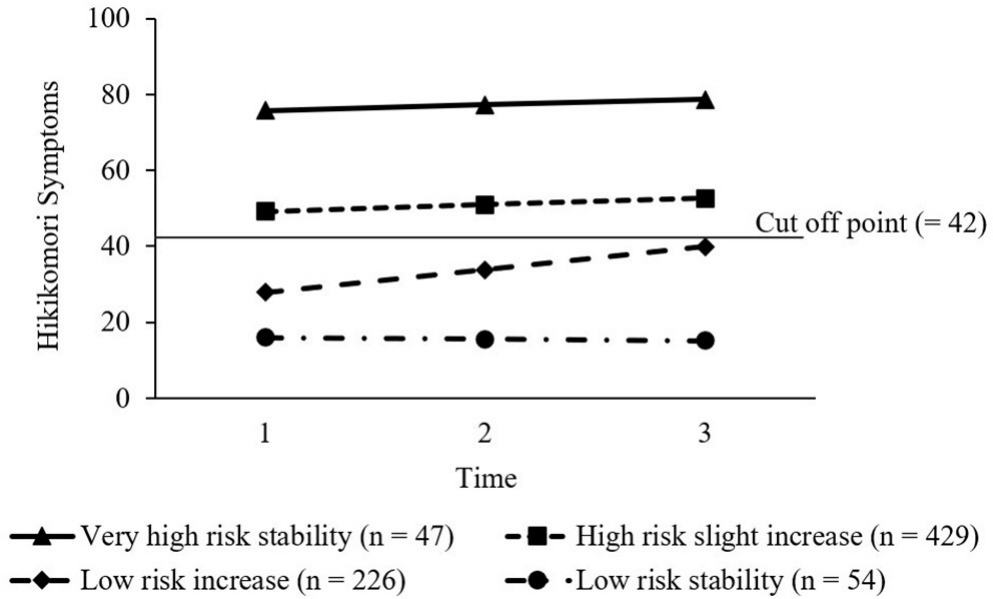
The four trajectories of hikikomori symptoms.

To extract the trajectories of hikikomori symptoms, we compared the results of one-to six-class models. Based on the fit indices for the different models reported in Table 4, we found that the four-class model was superior to other models. The entropy was acceptable for three-to six-class models. Adding a fourth class improved LMR-LRT, whereas adding a fifth class did not. The SSA-BIC value of the four-class model was lower than that of the three-class model. To test its stability, we performed double-split cross-validation. Results confirmed that similar four-class models were extracted in both subsamples. The class assignment for the entire sample was highly consistent for each of the two subsamples (κs = 0.79 and 0.93). Table 5 shows the growth parameters for the four-class model, and Figure 1 shows the four different trajectories.

**Table 4:** Model fits statistics for latent class growth analyses.

**Class solutions**	**SSA-BIC**	**Entropy**	**LMR-LRT**	**LMR-LRT** ***p*****-values**
One class	10,200.022	—	—	—
Two classes	10,046.891	0.549	155.660	<0.001
Three classes	9,876.699	0.787	171.904	<0.001
**Four classes**	**9,838.513**	**0.737**	**46.220**	**0.002**
Five classes	9,824.865	0.721	22.856	0.074
Six classes	9,806.018	0.747	27.807	0.266

*SSA-BIC, sample size adjusted Bayesian information criterion; LMR-LRT, Lo-Mendell-Rubin likelihood ratio test; the preferred model is highlighted in bold*.

**Table 5:** Growth parameters for the final four-class model.

**Variables**	**Very high** **risk stability**	**High risk** **slight increase**	**Low risk** **increase**	**Very low** **risk**
	***M*** **(*SE*)**	***M*** **(*SE*)**	***M*** **(*SE*)**	***M*** **(*SE*)**
Intercept	75.71 (2.53)^***^	49.18 (0.69)^***^	27.82 (1.46)^***^	15.99 (1.91)^***^
Slope	1.49 (1.78)	1.75 (0.48)^***^	6.03 (1.35)^***^	−0.40 (1.51)
Mean percentage of times that the hikikomori score exceeded the cutoff point	100.00	80.03	6.27	0.00

*M, mean; SE, standard error*.

*** *p < 0.001*.

Based on the nature of the trajectories and the cutoff score proposed by a previous study (5), the four trajectories were labeled as *very high-risk stability* (*n* = 47; 6.22%), marked by very severe initial levels above the cutoff point and subsequent stability; *high-risk slight increase* (*n* = 429; 56.75%), characterized by a relatively severe initial level than the cutoff score with slightly escalating severity; *low-risk increase* (*n* = 226; 29.89%), marked by relatively low initial levels below the cutoff but worsening symptoms to near the cutoff; and *low-risk stability* (*n* = 54; 7.14%), characterized by weak symptoms below the cutoff score.

### Roles of Identity Distress on Trajectories of Hikikomori Symptoms

Table 6 reports the ORs for the association of various risk factors at T1 (identity distress, depressive symptoms, life satisfaction, career expectations, and gender) and the four trajectories of hikikomori symptoms, with each of the four trajectories as the reference group. Young adults with more severe identity distress were more likely to follow the *very high-risk stability* and *high-risk slight increase* trajectories than the *low-risk increase* and *low-risk stability* trajectories after controlling other predictors. The ancillary analysis showed that identity distress positively predicted the percentage of time in which the participants' hikikomori symptom scores exceeded the cutoff (β = 0.09, *p* = 0.013), after controlling for depressive symptoms (β = 0.29, *p* < 0.001), life satisfaction (β = −0.13, *p* < 0.001), career expectations (β = −0.15, *p* < 0.001), and gender (β = −0.09, *p* = 0.004).

**Table 6:** Analyses of multinomial logistic regression on trajectories of hikikomori symptoms.

**Trajectories**	**Reference group**	**Identity distress**	**Depressive** **symptoms**	**Life satisfaction**	**Career** **expectation**	**Gender** **(0 = men;** **1 = women)**
		**OR [95% CI]**	**OR [95% CI]**	**OR [95% CI]**	**OR [95% CI]**	**OR [95% CI]**
Very high risk stability	High risk slight increase	1.07 [1.00, 1.16]	1.04 [1.00, 1.07]	0.88 [0.83, 0.92]^***^	0.94 [0.86, 1.03]	1.42 [0.60, 3.39]
	Low risk increase	1.12 [1.03, 1.21]^**^	1.12 [1.08, 1.17]^***^	0.84 [0.79, 0.90]^***^	0.85 [0.76, 0.94]^**^	0.66 [0.25, 1.71]
	Low risk stability	1.15 [1.04, 1.27]^**^	1.17 [1.10, 1.24]^***^	0.79 [0.73, 0.85]^***^	0.82 [0.71, 0.94]^**^	0.57 [0.17, 1.88]
High risk slight increase	Low risk increase	1.04 [1.00, 1.08]^*^	1.09 [1.06, 1.11]^***^	0.96 [0.94, 0.99]^*^	0.90 [0.85, 0.96]^**^	0.46 [0.29, 0.74]^**^
	Low risk stability	1.07 [1.00, 1.15]	1.13 [1.07, 1.19]^***^	0.90 [0.85, 0.95]^***^	0.87 [0.78, 0.97]^*^	0.43 [0.17, 0.94]^*^
Low risk increase	Low risk stability	1.03 [0.96, 1.10]	1.04 [0.99, 1.09]	0.93 [0.88, 0.99]^*^	0.97 [0.87, 1.08]	0.87 [0.37, 2.04]

*OR, odds ratio; 95% CI, 95% confidence interval*.

* *p < 0.05*,

** *p < 0.01*,

*** *p < 0.001*.

Regarding other predictors, young adults with severe depressive symptoms were more likely to follow *very high-risk stability* and *high-risk slight increase* trajectories than *low-risk increase* and *low-risk stability* trajectories. Meanwhile, young adults with higher levels of life satisfaction were less likely to manifest *very high-risk stability* and the *high-risk slight increase* trajectories than the *low-risk increase* and *low-risk stability* trajectories. They were also less likely to follow the *low-risk increase* trajectory than the *low-risk stability* trajectory. Furthermore, young adults with higher career expectations were less likely to follow *very high-risk stability* and *high-risk slight increase* trajectories than *low-risk increase* and *low-risk stability* trajectories. Finally, men were more likely to show *high-risk slight increase* trajectories than *low-risk increase* and *low-risk stability* trajectories.

## Discussion

This study aimed to: (a) identify diverse trajectories of hikikomori symptoms among young adults who conduct job searches; and (b) examine whether identity distress predicts these trajectories while controlling for other risk factors. Through these examinations, we intended to gain a novel understanding of how and why young adults exhibit (or prevent) hikikomori symptoms, considering the developmental factors in this period that have been neglected in empirical studies (21).

Regarding the trajectories of hikikomori symptoms, in line with our hypothesis, we identified a small number of young adults who followed the *very high-risk stability* trajectory. Their scores on hikikomori symptoms exceeded the cutoff point at all time points. Young adults with this trajectory have difficulty finding relief from severe hikikomori symptoms, probably because they receive less support from social relationships. Furthermore, consistent with our hypothesis, we found two trajectories marked by exacerbated hikikomori symptoms. Specifically, the largest and second-largest groups were the *high-risk slight increase* and *low-risk increase* trajectories. These results may reflect that it is challenging for young adults in Japan to prevent hikikomori symptoms during the job search period. In contemporary Japan, many young adults engage in job searching under high pressure and unstable contexts, such as economic stagnation and casualization of employment (16–18). To avoid such a stressful situation, many young people in Japan may exhibit hikikomori symptoms and escalate them.

Contrary to our hypothesis, only a small proportion of young adults followed a *low-risk stability* trajectory, and a trajectory of modifying hikikomori symptoms was not found. Taken together with the finding that most young adults aggravated their hikikomori symptoms, the results emphasize that it is difficult for most young adults to prevent hikikomori symptoms to a certain degree during a job search. These are somewhat consistent with previous findings of epidemiological surveys conducted by the Cabinet Office of the Government of Japan, which suggest the escalation of the prevalence of hikikomori across the 2010s (9–11). The present study added these previous findings as it implies that the period of job search is significant for maintenance or escalation of severe hikikomori within each individual and is appropriate for providing interventions for many young adults. Furthermore, it is worth considering the impact of COVID-19 pandemic. Amid the COVID-19 pandemic, Japan has been hit with an economic depression similarly to other countries. Many jobs have been lost and young people are assumed to experience challenges and distress in searching for a job during the pandemic. Indeed, suicide rates of Japanese people increased in 2020 compared to those in 2016–2019, and this rate of increase was particularly pronounced among young people aged 30 years or younger (51). Additionally, Japan had non-coercive (mild) lockdowns for a certain period of time in 2020 (52). Some items of the HQ-25 (e.g., “I spend most of my time at home”) may reflect preferences and behaviors related to COVID-19 social isolation. Hence, there is a possibility that participants showed relatively severe levels of hikikomori symptoms and their escalation at T2 and T3 (May 2020 and November 2020). In fact, psychiatrists assume that those having a risk of hikikomori may choose not to participate in society and exhibit hikikomori symptoms after the COVID-19 pandemic period (2). While the present study cannot assess the impact of the pandemic on hikikomori symptoms, it is important to further explore trajectories of hikikomori symptoms including after the pandemic period.

As for risk factors, young adults with severe identity distress showed trajectories marked by severe hikikomori symptoms beyond those of other risk factors. Furthermore, their hikikomori symptom scores were likely to exceed the cutoff. These results align with our hypothesis, indicating the significance of identity in adolescence and young adulthood. Healthy identity helps young adults navigate their transition to the labor market in the challenging context of contemporary Japan (18, 53). Our results revealed that resolving identity distress and addressing other risk factors provide an important foundation for preventing hikikomori symptoms and promoting smooth social participation. This finding provides practitioners with novel insights into intervention strategies that would work for each hikikomori symptom pathway (54).

Despite the unique link of identity distress to hikikomori symptoms, it is also important to note that depressive symptoms showed stronger correlation with (*r* = 0.51) and prediction for (β = 0.29) hikikomori symptoms, compared to identity distress. These results are congruent with prior findings that depression is one of the most critical disorders that are related to hikikomori symptoms (6, 7, 19). Given that identity distress is closely related to depressive symptoms (31), young adults with strong and prolonged identity distress may have high levels of depressive symptoms and lead to extreme hikikomori symptoms.

Taken together with a series of findings, this study suggests that group interventions for solving severe identity distress may be effective for primary prevention of hikikomori. We found that many young adults experienced severe and exacerbated hikikomori symptoms during their job search. Others interacting with adolescents and young adults are less likely to notice their hikikomori symptoms at an early stage (3). Thus, it may be useful to provide preventive group-based interventions for modifying identity distress during job search, not restricted to few specific young people who manifest severe hikikomori. To modify identity distress, it is necessary to help young people construct a meaningful connection across past, present, and future views of self and acquire clear understandings of what role they play in society (25, 26). These processes proceed through conversations or discussions with responsive others (55). In these conversations, young people narrate their past experiences and receive questions that elaborate their reflection and self-understanding from responsive listeners (e.g., “how did you feel about that?”) (56). Based on such elaborative scaffolding, young people make meaning from their experiences, and clarify how their selves are interwoven across time and how these selves are related to sociocultural expectations. Given that young people with high risk of hikikomori are likely to experience negative events (e.g., bullying in school) (2), it is important to promote their positive meaning making for negative experiences. Hence, group-based interventions that provide young people with opportunities to discuss about their past experiences with responsive others may contribute to hikikomori prevention. Indeed, a prior study showed that a group-based intervention encouraging young adults' self-understanding and identity exploration through discussion with group members reduced their identity distress (57). It is effective to apply such exercises to group counseling in the student counseling room or university classes on career guidance (58). Furthermore, for young adults who hesitate to attend a face-to-face discussion, an interactive text-based intervention may be effective (59). This approach may be useful in the current situation of the COVID-19 pandemic.

Focusing on identity may compensate existing group-based interventions for hikikomori. In the past several decades, many schools in Japan have conducted interventions such as behavioral activation program (60) and mental health education (61) which are intended to prevent mental health problems such as depression. Furthermore, many schools have employed group-based interventions to promote young adults' job skills or techniques for job meeting (62). Additionally, researchers and practitioners have provided group-based social skills training to people with hikikomori and organizational support to their family members (1, 2). However, these interventions have not been intended to promote making meaningful connections between views of self across time or aligning their identity with sociocultural expectations. Hence, existing interventions may not work successfully for identity distress. In contemporary Japan, there have been social (the collapse of a seniority wage system and an increase in temporal employment) and cultural (the change from collectivism to individualism) shifts during the past decades (16, 17). These changes have rendered many young Japanese people unable to rely on models from older generations and re (enter) the labor market, and hence their identity development has become more difficult (63). Therefore, we believe that group-based interventions for modifying identity distress are required, especially in contemporary Japan.

As we mentioned above, group-based preventive interventions for reducing identity distress are suitable for school contexts. Indeed, Ministry of Education, Culture, Sports, Science, and Technology of Japan emphasizes that promoting students' successful self-actualization in society is an important goal of Japanese education (64), and this process is supported by healthy identity development (33). Meanwhile, the Japanese government recently appointed Mr. Tetsushi Sakamoto to the minister of loneliness to address mental health issues including hikikomori. Findings in the present study suggest the possibility that the minister of loneliness can cooperate with the Ministry of Education, Culture, Sports, Science and Technology and work to prevent hikikomori symptoms through interventions that promote healthy identity development.

The present study has several strengths. It reveals diverse trajectories of hikikomori symptoms, uses a longitudinal dataset, and provides novel knowledge about how identity distress predicts hikikomori trajectories. However, this study had several limitations that warrant further discussion. First, this study measured hikikomori symptoms using a self-report questionnaire. Future research should examine whether our results can be replicated using semi-structured interviews for diagnosis. Second, our data collection unexpectedly included the COVID-19 pandemic period. As restrictions on outings in the COVID-19 pandemic may have affected hikikomori symptoms among youth (2), it is important to conduct a replication study when the pandemic is over. Third, it is important to include other predictive factors in future research. For example, a previous study indicated that negative family relationships and overuse of the internet are related to hikikomori (15). Future research needs to examine whether identity distress predicts hikikomori symptoms after controlling these other factors. Finally, our longitudinal data were marked by low participant retention rates, which may be due to several reasons. For example, the small reward for answering the questionnaires (approximately 0.50 USD) did not increase participants' motivation. Future research should offer increased compensation for participation to increase the retention rate. Another reason, our online questionnaires included many items that impose psychological burden on participants (e.g., hikikomori symptoms, identity distress, and depressive symptoms). There is a possibility that participants who feel psychological distress in COVID-19 pandemic situation hesitated to answer these items. To increase participants' motivation to answer the questionnaires, it may be useful to decrease the number of items assessing psychological distress and minimize psychological burden of participants.

## Conclusion

In conclusion, the present study expands knowledge by revealing the heterogeneity of hikikomori trajectories through job search. The period of job search is a period of vulnerability in contemporary Japan, because many young adults experience difficulties during the transition to the labor market. This is the first study to demonstrate that many young adults show severe levels and an escalation of hikikomori symptoms over time, whereas a small portion of young adults prevent hikikomori symptoms during this period. This study also addressed this research gap by showing the unique role of identity distress in the trajectories of hikikomori symptoms. These findings suggest the importance of group-based interventions that promote identity development for preventing hikikomori symptoms. This study suggests novel avenues for further effective interventions to prevent and remediate hikikomori symptoms.

## Data Availability Statement

The datasets presented in this study can be found in online repositories. The names of the repository/repositories and accession number(s) can be found below: https://osf.io/afctj/.

## Ethics Statement

The studies involving human participants were reviewed and approved by the Ethics Review Board of Hiroshima University. The patients/participants provided their written informed consent to participate in this study.

## Author Contributions

SH developed the research design, collected data, and wrote the first draft of the manuscript. SH performed statistical analyses with KK and TU. KK, TU, KH, and KS critically revised the manuscript. All authors contributed to data interpretation, have reviewed and approved the submission of this manuscript.

## Funding

This study was supported by a Japan Society for the Promotion of Science Grant-in-Aid for Young Scientists [Grant Number 20K14161].

## Conflict of Interest

The authors declare that the research was conducted in the absence of any commercial or financial relationships that could be construed as a potential conflict of interest.

## Publisher's Note

All claims expressed in this article are solely those of the authors and do not necessarily represent those of their affiliated organizations, or those of the publisher, the editors and the reviewers. Any product that may be evaluated in this article, or claim that may be made by its manufacturer, is not guaranteed or endorsed by the publisher.
